# miR-34a and miR-125b Expression in HPV Infection and Cervical Cancer Development

**DOI:** 10.1155/2015/304584

**Published:** 2015-06-09

**Authors:** Joana Ribeiro, Joana Marinho-Dias, Paula Monteiro, Joana Loureiro, Inês Baldaque, Rui Medeiros, Hugo Sousa

**Affiliations:** ^1^Virology Service, Portuguese Institute of Oncology of Porto, Rua Dr. António Bernardino de Almeida, 4200-072 Porto, Portugal; ^2^Molecular Oncology and Viral Pathology Group (CI-IPOP), Portuguese Institute of Oncology of Porto, Rua Dr. António Bernardino de Almeida, 4200-072 Porto, Portugal; ^3^Faculty of Medicine, University of Porto (FMUP), Alameda Prof. Hernâni Monteiro, 4200-319 Porto, Portugal; ^4^Abel Salazar Institute for the Biomedical Sciences (ICBAS), University of Porto, Rua de Jorge Viterbo Ferreira No. 228, 4050-313 Porto, Portugal; ^5^Department of Pathology of Portuguese Institute of Oncology of Porto, Rua Dr. António Bernardino de Almeida, 4200-072 Porto, Portugal; ^6^Research Department, Portuguese League against Cancer (Liga Portuguesa Contra o Cancro-Núcleo Regional do Norte), Estrada Interior da Circunvalação 6657, 4200-177 Porto, Portugal

## Abstract

We aimed to characterize miR-125b and miR-34a expression in 114 women with different cervical lesions: normal epithelium with (*n* = 20) and without (*n* = 29) HPV infection; LSIL (*n* = 28); HSIL (*n* = 29); and ICC (*n* = 8). miRNA expression analysis was performed by comparing the distinct groups with the reference group (women with normal epithelium without HPV). For miR-125b, we observed a twofold (2^−ΔΔCt^ = 2.11; *P* = 0.038) increased expression among women with normal epithelium with HPV infection and a trend of downregulation in different cervical lesions including an 80% reduction (2^−ΔΔCt^ = 0.21; *P* = 0.004) in ICC. Similarly, miR-34a expression analysis revealed an increased expression (2^−ΔΔCt^ = 1.69; *P* = 0.049) among women with normal cervix and HPV infection, and despite no significant correlation with cervical lesions, its expression was increased by twofold (2^−ΔΔCt^ = 2.08; *P* = 0.042) in ICC. Moreover, miR-125b levels were able to predict invasive cancers with 88% sensitivity and 69% specificity. Results showed that while miR-34a expression seems to be correlated with invasive cervical cancer, miR-125b expression is significantly changed within the different cervical lesions and their levels should be further investigated as possible predictive/prognostic biomarkers using a noninvasive approach.

## 1. Introduction

Cervical cancer is the third most common cancer among women with approximately 530 000 new cancer cases and 275 100 deaths each year [1, 2]. Persistent infection by human papillomavirus (HPV) has been considered the etiological cause of squamous intraepithelial lesions of the cervix that may develop into high-grade dysplasia or to invasive carcinoma. The majority of HPV infections are asymptomatic and are efficiently controlled by the host immune system; therefore, the outcome of HPV infection is variable [3]. High-risk HPVs (HR-HPV) are recognized to be a necessary but nonsufficient condition for the development of cervical cancer and clinicians are still demanding for the identification of useful predictive/prognostic biomarkers for HPV infection [4–7].

Recently, microRNAs (miRNAs), noncoding RNAs with approximately 18–25 nucleotides in length, have been proposed as biomarkers of cancer development. miRNAs are responsible for modulating gene expression by binding to complementary segments present in the untranslated region (UTR) of messenger RNA (mRNA) leading to the suppression of translation and/or degradation of mRNA [8]. miRNAs are thought to potentially target up to one-third of human coding genes managing cellular activity, including proliferation, differentiation, and apoptosis [8, 9]. These molecules have been described as key regulators in cancer, and, in fact, several studies have been addressing the impact of miRNAs in tumor development either by acting as oncogenes or tumor suppressor genes [9, 10].

Several studies have attempted to identify potential biomarkers of HPV infection outcome by studying the events of HPV-related carcinogenesis [11, 12]. Recently, it was suggested that some miRNAs could be biomarkers for the occurrence and development of the HPV-associated cancers, including cervical cancer [13]. Moreover, studies have described several interactions between miRNAs and HPV oncoproteins and specific miRNAs have been located in cancer-related genomic regions, which include fragile sites at or near HPV integration sites. Therefore, the identification of different tumor-specific miRNA signatures might be an important tool to distinguish the different HPV-associated lesions or cancers [14–16].

The aim of this study was to characterize the expression of two miRNAs (miR-34a and miR-125b) in cytological samples from women with different cervical lesions, including invasive cervical cancers, and evaluate its impact as predictive/prognostic biomarkers of cervical cancer and HPV infection.

## 2. Subjects, Materials, and Methods

### 2.1. Study Population

The study was performed in exfoliated cervical cells collected from randomly selected women (*n* = 114, median age 40 ± 12.6 years old) attended at the Gynaecological Service from the Portuguese Institute of Oncology of Porto (IPO Porto) during routine clinical observations. These women are followed up in our institution due to previous history of cancer (not specifically cervical cancer). All samples were submitted to cytological examination and classified according to the Bethesda classification by qualified pathologists from our institution: 49 women with normal cytology, 28 with low-grade intraepithelial squamous lesions (LSIL), 29 with high-grade intraepithelial squamous lesions (HSIL), and 8 with invasive cervical carcinomas (ICC).

### 2.2. Sample Processing

Samples were collected in* ThinPrep* tubes (Hologic, USA) and stored at room temperature prior to processing: a 4 mL aliquot was used for* digene HC2 High-Risk HPV DNA Test* (QIAGEN, Germany); and 1 mL was used for total nucleic acids extraction using* High Pure Viral Nucleic Acid Kit* (Roche, Germany). DNA/RNA was quantified using the NanoDrop 1000 Spectrophotometer v3.7 (Thermo Scientific, Wilmington, DE, USA).

### 2.3. HPV Status

HPV was detected at the Virology Service of IPO Porto as part of routine procedures using* digene HC2 High-Risk HPV DNA Test* (QIAGEN, Germany) (HC2). HC2 test detects 13 high-risk HPV (HR-HPV) types: 16, 18, 31, 33, 35, 39, 45, 51, 52, 56, 58, 59, and 68. HPV infection was detected in 67.3% of all cervical specimens, with a prevalence of 40.8% (20/49) for normal cytology, 75.0% (21/28) for LSIL, 96.6% (28/29) for HSIL, and 87,5% (7/8) for ICC.

### 2.4. miRNA Analysis

miR-125b, miR-34a, and miR-23a were analysed using two-step real-time PCR protocols with TaqMan MicroRNA Assays: hsa-miR-125b-5p_000449; hsa-miR-34a-5p_000426; hsa-miR-23a-3p_000399 (Applied Biosystems, Foster, CA, USA). Reverse transcriptase reactions were performed using TaqMan MicroRNA Reverse Transcription Kit (Applied Biosystems, Foster, CA, USA) in a 15 *μ*L of total volume reaction mix with 7 *μ*L of master mix containing 1x RT buffer, 1.0 mM of total dNTPs, 50 U MultiScribe Reverse Transcriptase Enzyme, and 0.25 U of RNase inhibitor; 3 *μ*L of RT primers (Applied Biosystems, Foster, CA, USA); and 5 *μ*L of RNA sample. The amplification conditions were as follows: annealing at 16°C for 30 min, extension at 42°C for 52 min, and RT inactivation at 85°C for 10 min. All reverse transcriptase reactions included two nontemplate controls using double distilled water to replace template RNA. qPCRs were performed on Applied Biosystems 7300 Real-Time PCR System (Applied Biosystems, Foster, CA, USA) with a 20 *μ*L final volume mixture containing 1x TaqMan Universal PCR Master Mix (Applied Biosystems, Foster City, California, USA), 1x MicroRNA Assay (Applied Biosystems, Foster City, California, USA), and 2 *μ*L cDNA (RT product). Thermal cycling conditions were 10 min. at 95°C followed by 45 cycles of 15 sec. at 95°C and 1 min. at 60°C.

### 2.5. Data Analysis

The endpoint of qPCR data is the threshold cycle (Ct), which represents the fractional cycle number at which the fluorescence reaches the fixed threshold. All qPCR reactions were run in duplicate and all experimental replicates had less than 0.99 Ct difference.

The relative quantification of miRNA expression was analyzed using Livak method (also known as 2^−ΔΔCt^ method). In this method, Cts from the target miRNAs (miR-125b and miR-34a) in both test and reference cases were adjusted in relation to the Ct of a normalizer miRNA (miR-23a) resulting in ΔCt. The reference group in this study was women with normal cytology and without HPV infection. The difference between the ΔCt of cases and ΔCt reference gives the ΔΔCt value, which is incorporated to determine the fold-difference in expression. The range of fold-differences was also calculated for each type of lesion.

### 2.6. Statistical Analysis

Statistical analysis was performed using the computer software* IBM SPSS (Statistical Package for Social Sciences) Statistics* version 20.0 for Mac. All groups were compared, whenever appropriate, by Student's *t*-test, ANOVA, and Chi-square (*χ* ^2^) test. Results are considered to be significantly different when *P* < 0.05. Receiver operating characteristic (ROC) curves were used to assess the predictability of cervical disease progression.

## 3. Results

### 3.1. qPCR Analysis

**Figure 1:**
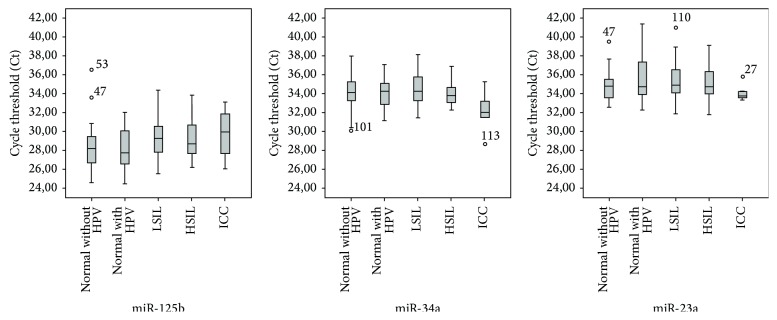
Cycle threshold of analysed miRNAs. LSIL: low-grade squamous intraepithelial lesions; HSIL: high-grade squamous intraepithelial lesions; ICC: invasive cervical carcinoma.

qPCR results are shown in Table 1 and Figure 1. Firstly, we noticed that qPCR was able to detect both low and high quantities of the miRNAs (overall Ct values range was 24.53 to 41.35). Additionally, the variation coefficient (VC) for all miRNAs was below 10%, which reveals that the variation of Ct within samples was very low. These data also show that the* High Pure Viral Nucleic Acid Kit* (Roche, Germany) was able to efficiently extract miRNA from this type of samples.

### 3.2. miRNAs Expression

Expression of selected miRNAs was determined for all samples, grouped according to cytological result, using women with normal cytology and without HPV infection as control group. Firstly, we have tested the influence of the presence of HPV in the different cervical lesions in data analysis; nevertheless, the results showed no statistically significant differences (data not shown).

According to the Livak method, the ratio between the 2^−miR23a  Average  Ct  of  controls^ and 2^−miR23a  Average  Ct  of  all  cases^ of miR-23a revealed no significant difference on the overall data regarding the miR-23a distribution in all subgroups (ratio = 1.18) [17]. Moreover, the *t*-test showed no statistically significant differences between the distribution of miR-23a in the two groups (*P* = 0.555). Therefore, we have considered that miR-23a could be used as the normalizer of the analysis of miR-125b and miR-34a expression.

**Figure 2:**
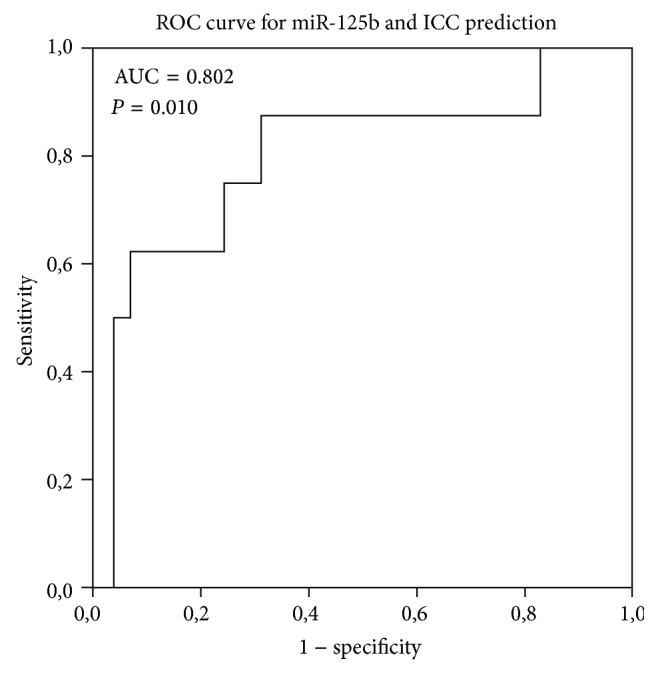
Prediction of ICC development using miR-125b levels. AUC: area under the curve.

Considering the results of miR-125b expression (Table 2) there are interesting data: firstly, we observed a twofold (2^−ΔΔCt^ = 2.11; *P* = 0.038) increase in the group of women with normal cytology with HPV infection; then, despite not being statistically significant, we also found a decrease of miR-125b expression for LSIL or HSIL (2^−ΔΔCt^ = 0.75 and 2^−ΔΔCt^ = 0.55, resp.); and, finally, the analysis revealed that miR-125b expression was significantly decreased in women with ICC (2^−ΔΔCt^ = 0.21; *P* = 0.004). The accuracy of miR-125b levels to discriminate between the different cervical lesions and normal cases was evaluated using ROC curves; nevertheless, significant results were only observed for ICC where the cut-off of −5.8 (ΔCt value) allows discriminating ICC with 88% sensitivity and 69% specificity (area under the curve = 0.802; *P* = 0.010) (Figure 2).

Similar to miR-125b, miR-34a expression in women with normal cytology and with HPV infection was also significantly different from the control group (2^−ΔΔCt^ = 1.69; *P* = 0.049) and women with ICC (2^−ΔΔCt^ = 2.08; *P* = 0.042) (Table 2). Despite these differences and in contrast to miR-125b, there was no trend for miR-34a expression in cervical lesions as the results showed that it seems to be stably expressed in LSIL and HSIL (2^−ΔΔCt^ = 1.01 and 2^−ΔΔCt^ = 1.10, resp.).

## 4. Discussion

Actually, miRNA expression and biological functions are highly influenced by cellular context, probably due to the differential expression of their target mRNAs [9]. In fact, the miRNAs expression varies from tissue to tissue and several studies proved that they are differentially expressed in abnormal tissues when compared with normal adjacent tissues and therefore have been described as possible biomarkers of cancer development. Moreover, miRNAs are thought to be highly stable when compared with mRNA and, therefore, their detection is relatively easy and reproducible, suggesting they could be used as good candidates for biomarkers and diagnostic tools in oncology [9]. However, it is important to consider that microRNA expression can be affected by several factors such as the type of treatment that the patients are submitted to. Recent studies have been developed to identify proof-valuable biomarkers for HPV infection outcome considering the cellular modifications caused by HPV infection and the cascade of distinct events of HPV carcinogenesis [11]. Literature has shown differential miRNA expression in cervical lesions and cancer tissues and also revealed potential interactions of miRNAs in HPV-associated carcinogenesis [13]. In our study, we investigated the expression of two miRNAs (miR-125b and miR-34a) that have been described as interacting in crucial steps of cervical cancer development.

Our study is the first using qRT-PCR methodology to evaluate the expression of miRNAs in exfoliated cervical cells with high sensitivity and specificity. Nevertheless, we are aware that our study has some limitations such as the following: the possibility of potential selection bias cannot be ruled out since the study population was hospital-based; the limited number of samples, mainly in ICC group, may have some impact on the precision of data and, therefore, it would be useful to develop a further study with increased number of samples; the type of sample used, cervical exfoliated cells, is not the most frequently used in these types of studies, although clinicians frequently request the use of noninvasive techniques for the research of new biomarkers; therefore further studies should be performed, especially in matched cytology-histology samples, to evaluate the correlation between the specificity/sensitivity of the analysis; and, finally, the fact that we have not used a specific kit for miRNA extraction could have some impact on data quality. Although the use of TaqMan assays provides feasibility to the study and considering that the specificity is extremely high, the exclusion of DNA amplification would be an important factor to address since we have not used a specific kit for miRNA extraction. Nevertheless, regarding this last point, in our study, the amplification of reference miRNA has shown very good coefficient of variation (<7%). Moreover, in RT-qPCR methodology, data normalization is crucial to obtain accurate results. According to Shen et al., who analysed the stability of candidate miRs and small RNA reference genes in cervical cancer cell lines and other cervical specimens, miR-23a has proven to be optimal reference microRNA and was suggested to be used for normalization [18]. In our study, the data considering miR-23a also revealed that this microRNA is a good endogenous reference to cervical samples.

miR-125b has been described to be involved in different cellular processes such as inflammation, cell proliferation, and cell cycle regulation and, therefore, it can act as oncogene or tumor suppressor depending on cell type [19–22]. We demonstrated that miR-125b expression is increased in normal cervix infected with HPV, while the relative expression seems to decrease as lesions progress (Table 2). According to literature, an increased expression of miR-125b shortly after HPV infection can be explained by interaction with viral proteins [23, 24]. Based on a recent study that revealed strong homology between miR-125b and the HPV-L2, an increased expression of miR-125b is a quick response to HPV productive infection shortly after viral infection. Nuovo and colleagues showed that after persistent infection, HPV-L2 can induce inactivation of miR-125b and this event seems to be associated with cytological changes of the koilocytes [25]. Although literature refers to the fact that miR-125b is a negative regulator of p53 and its expression would allow a more effective suppression of p53 pathways leading to tumor development [3, 20, 26, 27], it has been shown that overexpression of miR-125b leads to decreased cell proliferation, apoptosis, and suppression of tumor growth by targeting the PI3K/Akt/mTOR signaling pathway [28]. This evidence must be further studied to clarify what is promoting the underexpression of miR-125b in cervical carcinogenesis after HPV infection.

miR-34a is a key regulator of tumor suppression and has been reported to be downregulated in several cancers [29]. This miRNA is implicated in the p53 network and has been described as direct target of p53: when p53 is activated, it induces the transcription of miR-34a, which is able to target several molecules involved in cellular transformation and carcinogenesis [14, 15, 29–32]. Theoretically, in HPV-associated carcinogenesis, the expression of HPV-E6 will downregulate the expression of miR-34a by targeting p53 to degradation through the ubiquitin-proteasome system [26, 29, 33, 34]. Therefore, it is expected that miR-34a deregulation occurs after HPV infection, probably after viral genome integration into host's genome. Li and colleagues reported that inhibition of miR-34a expression is observed in precancerous lesions and probably represents an early onset event in cervical cancer development [29, 33]. Several studies have showed that the expression level of miR-34a is significantly reduced in both cancer tissues and cervical cell lines, compared with normal tissues [33, 35]. Although we have found an initial increase of miR-34a expression in normal cells infected by HPV, we did not observe significant differences in relative expression when considering LSIL and HSIL. The increase of miR-34a expression in HPV infected cells is probably explained by the activation of cellular repair mechanisms after viral infection that would activate p53 pathways and therefore induce miR-34a expression. Surprisingly, in our study, we observed a significant increase of miR-34a expression in women with invasive carcinomas, which increased the discussion on this subject since previous studies have shown controversial results regarding miR-34a and invasive cervical carcinoma [29, 33, 36]. Hence, it is important to perform more studies on miR-34a levels in HPV infection and cervical lesions/cancer and to clarify whether there are other pathways besides the p53-associated pathways, which are able to activate miR-34a expression in HPV-associated lesions.

In conclusion, our study revealed that both miR-125b and miR-34a are overexpressed in HPV infected cells and this should be further investigated to establish whether this is a direct consequence of viral infection or a cellular response. Additionally, our study revealed that miR-125b levels decrease as cervical lesions progress to invasive cervical cancer and ROC curve revealed that its levels could be used with good sensitivity and specificity in ICC diagnosis. Thus, being preliminary, these results suggest that miR-125b should be further investigated as a predictive/prognostic noninvasive tool of cervical cancer development.

## Figures and Tables

**Table 1:** qPCR data analysis.

	miR-125b	VC	miR-34a	VC	miR23a	VC
	Mean Ct ± sd		Mean Ct ± sd		Mean Ct ± sd	
Normal (HPV−) (*n* = 29)	28.43 ± 2.48	0.09	34.14 ± 1.82	0.05	34.94 ± 1.72	0.05
Normal (HPV+) (*n* = 20)	28.06 ± 2.20	0.08	34.09 ± 1.58	0.04	35.65 ± 2.58	0.07
LSIL (*n* = 28)	29.34 ± 2.12	0.07	34.60 ± 1.71	0.05	35.42 ± 2.04	0.06
HSIL (*n* = 29)	29.31 ± 2.13	0.07	34.02 ± 1.18	0.03	34.96 ± 1.70	0.05
ICC (*n* = 8)	29.78 ± 2.46	0.08	32.17 ± 1.90	0.06	34.02 ± 0.79	0.02

Ct: cycle threshold; Sd: standard deviation; VC: variation coefficient; LSIL: low-grade squamous intraepithelial lesions; HSIL: high-grade squamous intraepithelial lesions; ICC: invasive cervical carcinoma.

**Table 2:** Expression profile data for miR-125b and miR-34a in cervical lesions.

	miR-125b			miR-34a		
	ΔCt ± sd	2^−ΔΔCt^	*t*-test	ΔCt ± sd	2^−ΔΔCt^	*t*-test
Normal (HPV−) (*n* = 29)	−6.51 ± 1.73	Reference		−0.80 ± 1.16	Reference	
Normal (HPV+) (*n* = 20)	−7.59 ± 1.77	2.11 (0.64–7.01)	**0.038**	−1.56 ± 1.47	1.69 (0.76–3.78)	**0.049**
LSIL (*n* = 28)	−6.09 ± 1.44	0.75 (0.23–2.48)	0.320	−0.82 ± 1.81	1.01 (0.45–2.27)	0.955
HSIL (*n* = 29)	−5.65 ± 2.15	0.55 (0.17–1.83)	0.099	−0.94 ± 1.59	1.10 (0.49–2.46)	0.707
ICC (*n* = 8)	−4.24 ± 2.10	0.21 (0.06–0.69)	**0.004**	−1.86 ± 1.56	2.08 (0.93–4.66)	**0.042**

Ct: cycle threshold; Sd: standard deviation; LSIL: low-grade squamous intraepithelial lesions; HSIL: high-grade squamous intraepithelial lesions; ICC: invasive cervical carcinoma.
